# Cumulative associations between health behaviours, mental well-being, and health over 30 years

**DOI:** 10.1080/07853890.2025.2479233

**Published:** 2025-04-24

**Authors:** Tiia Kekäläinen, Johanna Ahola, Emmi Reinilä, Tiina Savikangas, Marja-Liisa Kinnunen, Tuuli Pitkänen, Katja Kokko

**Affiliations:** a Gerontology Research Center and Faculty of Sport and Health Sciences, University of Jyväskylä, Jyväskylä, Finland; b Laurea University of Applied Sciences, Vantaa, Finland; c The Wellbeing Services County of Central Finland, Jyväskylä, Finland; d School of Medicine, University of Eastern Finland, Kuopio, Finland; e Finnish Youth Research Society, Helsinki, Finland

**Keywords:** Metabolic health, mental health, depression, smoking, alcohol, physical activity, longitudinal, midlife, old age

## Abstract

**Background:**

Both the number of risky health behaviours and the duration of exposure to these behaviours over time may increase the risk of later adverse outcomes. This study examined cumulative associations of risky health behaviours with both positive and negative aspects of mental well-being and health. It has a uniquely long follow-up period of over 30 years, from early adulthood to the beginning of late adulthood.

**Materials and methods:**

The data were from the Jyväskylä Longitudinal Study of Personality and Social Development. The participants represent the Finnish age cohort born in 1959. This study utilized data collected at ages 27 (1986), 36 (1995), 42 (2001), 50 (2009), and 61 (2020–2021) (*n* = 206–326). Risk scores indicating the current number of risky behaviours of smoking, heavy alcohol consumption, and physical inactivity and their temporal accumulation over time were calculated. The associations of risk scores with mental well-being (depressive symptoms, psychological well-being) and health (self-rated health, number of metabolic risk factors) from age 36 onwards were analyzed with linear multilevel models adjusted for gender and education.

**Results:**

More current risky behaviours were associated with more depressive symptoms (*B* = 0.10, *p* = 0.032), lower psychological well-being (*B* = -0.10, *p* = 0.010), lower self-rated health (*B* = -0.45, *p* < 0.001), and more metabolic risk factors (*B* = 0.53, *p* = 0.013). The associations of temporal risk scores with the outcomes were even stronger (depressive symptoms: *B* = 0.38, *p* < 0.001; psychological well-being: *B* = -0.15, *p* = 0.046; self-rated health: *B* = -0.82, *p* < 0.001; metabolic risk factors: *B* = 1.49, *p* < 0.001). Among individual behaviours, the temporal risk score of alcohol consumption was negatively associated with most outcomes, while smoking was associated with poorer mental well-being and physical inactivity with poorer health.

**Conclusions:**

The current and temporal accumulation of multiple risky health behaviours were associated with poorer mental well-being and health. Preventing these behaviours early in adulthood and midlife is crucial to avoid their accumulation and subsequent health risks.

## Introduction

Noncommunicable diseases are the reason for 74% of all deaths globally, and risky health behaviours are the primary modifiable risk factors for them [1,2]. Risky health behaviours are actions taken by individuals that affect their health in a harmful way [3]. These include smoking, consuming alcohol heavily (e.g. beyond recommended limits of 8 portions per week for women and 15 for men [4]), and physical inactivity (usually defined as not achieving physical activity guidelines [5]. These three risky behaviours are well-known risk factors for multiple diseases, functional impairments, and premature death [1].

It is necessary to consider health behaviours together, as they are interrelated [6–8] and the significance of individual health behaviours depends on other health behaviours [9,10]. Even a single risky behaviour increases the risk of premature death and diseases, but together with other health behaviours, the impact is cumulative [9–13]. Furthermore, the impact of these behaviours on health accumulates throughout a lifetime [14]. An individual’s risk of later adverse outcomes increases with both the number of risky behaviours they engage in and the duration of exposure to these behaviours (temporal accumulation) over time [10,14]. For example, while smoking at age 50, 60, or 70 was associated with a higher risk of having reduced functional capacity at age 75, the risk increased further if smoking persisted at multiple points during these three measurements [15].

A limitation of previous literature is that the follow-up times have varied around 20 years ranging from early or mid-midlife to older age. It would be important to consider also early adulthood as most health behaviours are adapted before age 30 [16,17] and their effect may cumulate already years before midlife. From a life course perspective, it is crucial to understand whether risky health behaviours contribute to detrimental health outcomes already in midlife and the beginning of late adulthood, before older age. This understanding may provide new perspectives on methods and timing to promote healthy lifestyles and prevent health risks that occur later in old age.

While the cumulative associations of health behaviours with cognitive [10,11] and physical functioning [14,15] as well as mortality [9,12,18] are more studied, less information is available on mental well-being outcomes. Health behaviours and their combinations also predict mental well-being, such as life satisfaction and depression [19–23], but cumulative associations of health behaviours remain less clear. While mental well-being is a meaningful outcome in itself, it also contributes to public health [24]. For example, depressive symptoms increase the risk of frailty [25], physical multimorbidity [26], and premature mortality [27]. On the contrary, the positive aspects of mental well-being are resources protecting from diseases and mortality [24,28,29]. Thus, the cumulative associations of health behaviours with both physical and mental health outcomes should be considered also from a public health perspective.

The main gaps in the current literature are the need for longer follow-up periods beyond 20 years, particularly considering also early adulthood and the lack of information on mental well-being outcomes. This study contributes to this area of research by providing uniquely long follow-up over three decades from early adulthood to the beginning of late adulthood together with outcomes capturing both the positive and negative side of mental well-being as well as subjective and objective health. The present study aimed to investigate the associations of three risky health behaviours, namely smoking, heavy alcohol consumption, and physical inactivity, with mental well-being and health. Both combinations of risky health behaviours at one time point and their temporal cumulative association were investigated.

## Methods

### Participants

The data were from the Jyväskylä Longitudinal Study of Personality and Social Development (JYLS) [30,31]. JYLS was started in 1968 and the same participants, born in 1959, have been followed from age 8 to age 61. The initial sample (*N* = 369, 173 girls and 196 boys) comprised 12 randomly selected complete school classes from the area of Jyväskylä, Finland. For the present study, data collected at ages 27 (in 1986), 36 (in 1995), 42 (in 2001), 50 (in 2009) and 61 (from 2020 to 2021) were utilized.

In each adulthood data collection, the eligible participants were sent an information letter, a written informed consent form, and a life situation questionnaire. They were asked to fill in the consent form and life situation questionnaire and return them *via* mail. After that, consented participants were contacted by phone, and, depending on the participants’ consent, the psychological interview and/or health examination were scheduled. Psychological interviews consisted of structured and open-ended questions on various areas of life (e.g. work, family, human relationships, life events, personality). In the context of the interviews, the participants filled in several inventories (e.g. inventories for depressive symptoms and psychological well-being). The health examinations were part of the study from age 42 onwards and conducted by a physician and a nurse at ages 42 and 50, and by a nurse at age 61. The examination included an interview about medications, symptoms, diseases, and health in general, as well as basic assessments (blood pressure, heart rate, and anthropometric measurements). Participants were also invited to take laboratory tests (basic blood count).

**Figure 1.**
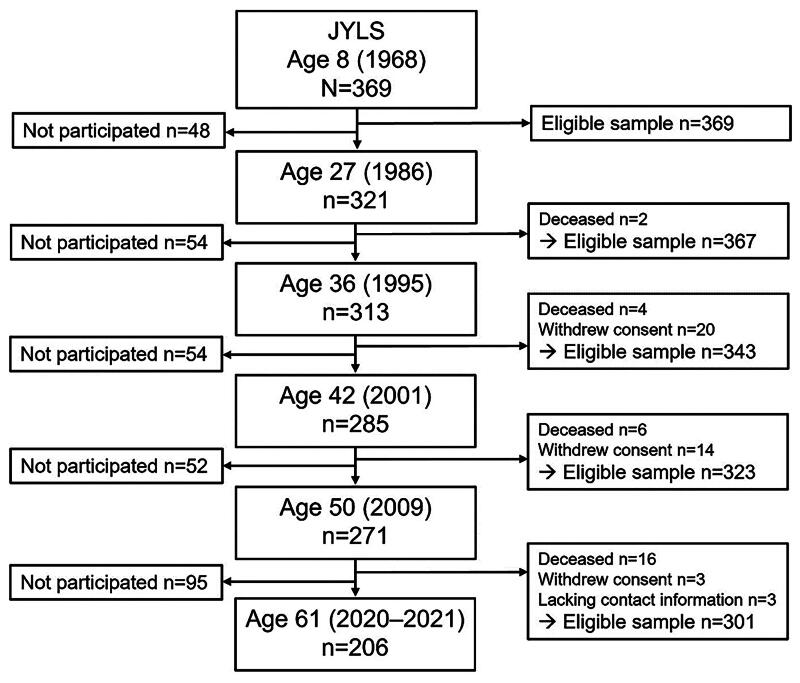
Flow chart of the participants for the present study.

Since the entire intended sample participated in the study in 1968, there was no initial attrition [31]. The participation rate has remained consistently high over the years [30,31], and the sample participating in the latest data collection at age 61 (*N* = 206) still represents both the initial sample and the respective Finnish age cohort relatively well [30]. The flow of the participants for the data collection waves used in the present study is shown in Figure 1.

The data collections have followed the ethical guidelines in place at the time [31]. In the adulthood data collections used in the present study, participants were asked for written informed consent in each data collection wave. The Ethical Committee of the Central Finland Health District approved the data collection at ages 42 (No. 42/2000) and 50 (No. 10E/2008) [32,33], and the latest data collection at age 61 was approved by the Ethical Committee of the University of Jyväskylä, Finland (13/2019) [30]. At ages 27 and 36 ethical approval was not relevant since no health examinations were conducted then.

### Measurements

Outcomes:

Outcomes included measures of mental well-being (depressive symptoms, psychological well-being) and health (self-rated health, metabolic health). Negative (depressive symptoms) and positive (psychological well-being) aspects of mental well-being and self-rated health were measured from age 36 onwards, and metabolic health measured from age 42 onwards.

Depressive symptoms were assessed with the depression scale of the General Behaviour Inventory [34]. It is a valid measure of depressive symptoms also in non-clinical populations [35]. The scale consisted of 16 items (e.g. “Have you become sad, depressed, or irritable for several days or more without really understanding why?”) with response options “1 = never”, “2 = sometimes”, “3 = often”, and “4 = very often”. The mean score was calculated (possible range 1–4) with a higher score indicating more depressive symptoms. The Cronbach’s alphas ranged from 0.89 to 0.93 at different data collection phases [36,37].

Psychological well-being was assessed with a shortened 18-item form of the Scales of Psychological Well-being [38]. The scale measures six components of psychological well-being, namely autonomy, environmental mastery, personal growth, positive relationships with others, purpose in life, and self-acceptance. The scale has good psychometric properties and captures key aspects of positive psychological functioning [38]. The response options were “1 = strongly disagree”, “2 = slightly disagree”, “3 = slightly agree”, and “4 = strongly agree”. The mean score of the 18 items was calculated (possible range 1–4) with a higher score indicating higher psychological well-being. The Cronbach’s alphas ranged from 0.72 to 0.79 at different data collection phases [37,39].

Self-rated health was assessed with a single question. The participants were asked to rate the question “How has your state of health been during the past year?” on a five-point Likert-type scale “1 = very good”, “2 = quite good”, “3 = moderate”, “4 = quite poor”, and “5 = very poor” (reversed for analysis, a higher score indicating better self-rated health).

Metabolic health was operationalized as five indicators: blood pressure (BP), waist circumference, triglycerides (TG), HDL cholesterol (HDL), and plasma glucose [40,41]. The number of metabolic risk factors was calculated following ATP III (Adult Treatment Panel III) [42]. The assessments are described in more detail in Supplementary Materials (Table S4). The number of risk factors ranged from 0–5 (0 = no risk factors to 5 = all five risk factors).

Risky health behaviours:

Smoking, heavy alcohol consumption, and physical inactivity were chosen for the present study as they were investigated in all adult data collection waves, that is, at ages 27, 36, 42, 50, and 61, with corresponding self-rated questions. The exact questions are presented in supplementary materials (Tables S1-S3).

**Table 1.** The associations of the number of current risky health behaviors (current risk score) and their accumulation over time (temporal risk score) with mental well-being and health

	Depressive symptoms		Psychological well-being		Self-rated health		Metabolic risk score	
	B	95% CI	B	95% CI	B	95% CI	B	95% CI
**Model 1**								
Current risk score	**0.10**	0.01–0.19	**−0.10**	−0.18– −0.02	**−0.45**	−0.67– −0.24	**0.53**	0.11–0.95
Gender	**0.11**	0.03–0.19	0.01	−0.05–0.08	−0.14	−0.30–0.01	**−0.53**	−0.85– −0.22
Education	**−0.07**	−0.11– −0.03	**0.08**	0.05–0.12	**0.10**	0.02–0.19	−0.11	−0.28–0.06
Age	0.01	−0.01–0.02	−0.01	−0.02–0.01	**−0.13**	−0.17– −0.10	**0.72**	0.63–0.80
**Model 2**								
Temporal risk score	**0.38**	0.20–0.56	**−0.14**	−0.29– −0.003	**−0.82**	−10.18– −0.46	**10.49**	0.74–20.25
Gender	**0.13**	0.04–0.21	0.02	−0.04–0.10	**−0.17**	−0.33– −0.002	**−0.47**	−0.79– −0.15
Education	**−0.05**	−0.10– −0.01	**0.07**	0.04–0.11	**0.09**	0.001–0.17	0.01	−0.16–0.18
Age	0.02	−0.001–0.03	−0.01	−0.02–0.004	**−0.12**	−0.16– −0.08	**0.74**	0.64–0.83
**Model 3**								
Current risk score	−0.05	−0.20–0.11	−0.07	−0.19–0.05	−0.08	−0.45–0.29	0.10	−0.65–0.85
Temporal risk score	**0.44**	0.18–0.70	−0.06	−0.27–0.15	**−0.73**	−10.29– −0.17	**10.39**	0.26–20.52
Gender	**0.13**	0.04–0.21	0.02	−0.04–0.09	**−0.17**	−0.33– −0.002	**−0.47**	−0.79– −0.15
Education	**−0.05**	−0.10– −0.019	**0.07**	0.04–0.11	**0.09**	0.002–0.17	0.01	−0.16–0.18
Age	0.02	−0.002–0.03	−0.01	−0.01–0.004	**−0.12**	−0.17– -0.08	**0.74**	0.64–0.84

Unstandardized estimates (B) and their 95% confidence intervals (CI) presented. Bolded estimates p < 0.05. Gender: Reference group men. Number of cases for the analysis with current risk score only / temporal risk score: N = 933/733 cases for depressive symptoms, N = 927/729 for psychological well-being, N = 987/823 for self-rated health, N = 541/390 for metabolic risk factors.

Smoking was assessed with questions concerning the frequency of smoking [6,43,44] and participants were coded either as 1 = current smokers (including both daily and occasional smokers) or 0 = nonsmokers (including never smoked or quit smoking). The question and response options were nearly identical for all ages (Table S1).

Alcohol consumption was assessed as the annual quantity of alcohol consumption in grams [6,45,46]. It was asked by using a quantity-frequency table which was the same at all ages except for adding some higher options at age 36 and again at age 42 (Table S3). The participants were coded either 1 = engaging in heavy alcohol consumption or 0 = not engaging in heavy alcohol consumption based on cut points of ≥ 7000 g of pure alcohol per year for women, and ≥ 10 000 g of pure alcohol per year for men. For participants who reported quitting alcohol consumption, a 1-year control period was required to account for heavy drinkers’ tendency for several short attempts to quit [45,46].

Physical inactivity was assessed with questions concerning the frequency of leisure-time physical activity [43,47]. The question was similar at ages 27 and 42–61 with slightly different response options at age 27 (Table S2). The exception was age 36 when the question concerned the extent of leisure time spent on physical activity. The participants were coded either as 1 = inactive (less than once a week, age 36: not at all) or 0 = active (physically active at least once a week, age 36: somewhat or mainly).

If possible, single missing values (i.e. a missing value for one risky behaviour at a given time point while the others are available) were imputed using other available information. For example, if smoking data was missing at age 50 and the participant reported at age 61 that they quit smoking after age 50, they were coded as current smokers at age 50.

Risk scores: We calculated three types of risk scores. First, *a current risk score* indicating the number of current risky behaviours a participant has (ranging from 0 = no current risky behaviours to 3 = smoking, heavy alcohol consumption, and physical inactivity). Second, *a temporal risk score* combining information on all three behaviours at each time point was calculated by summing up the current risk scores from the current and previous waves (e.g. a risk score at age 42 = number of risky behaviours at ages 27 + 36 + 42). Third, *a temporal risk score for each behaviour* separately for each time point was calculated (e.g. a sum score for smoking at age 50 = smoking 27 + smoking 36 + smoking 42 + smoking 50).

*The current risk score* was calculated only if a participant had information on at least two risky behaviours available. To account for possible missing information, the sum was divided by the number of available risky behaviour information (leading to a range of 0 = no current risky behaviours to 1 = having all three risky behaviours). A maximum of five cases per age phase had single missing values. *The temporal risk scores* were calculated for age 36 if a participant had information available from both ages 27 and 36. For the rest of the age phases (42, 50, and 61), the scores were calculated if a participant had missing information for a maximum of one measurement point prior. To account for possible missing waves, the sum was divided by the number of measurement points (leading to a range from 0 = no risk behaviours at any time point so far to 1 = having all three risky behaviours at all time points so far). There were 5 cases at age 42, 14 cases at age 50, and 33 cases at age 61 with one missing data point in the earlier data collections.

Confounders.

Gender (0 = men, 1 = women) and highest education attained in adulthood (1 = vocational courses or less, 2 = vocational school, 3 = vocational college or polytechnic (bachelor’s level), 4 = university) were used as confounders. We selected only these basic sociodemographic characteristics that may act as potential confounders for the associations between risky health behaviours and mental well-being and physical health outcomes while avoiding overadjustment with variables that may serve as mediators for the associations (e.g. body mass index) [48].

### Statistical analysis

Descriptive characteristics at each measurement time point are presented as frequencies (n) and percentages (%) or means (M) and standard deviations (SD). The associations of risky health behaviours with mental well-being and physical health outcomes were estimated with linear multilevel modelling. Multilevel models are suitable for longitudinal data as they take into account the nested structure of the data (i.e. the correlation between repeated measures on the same subjects over time) [49]. In addition, they benefit from using the maximum amount of data: for participants with missing data on outcomes in some measurement time points, the data from available time points are still included [49]. These models enabled the simultaneous investigation of the cumulative associations of multiple risky health behaviours over time [50].

Four sets of models were estimated, assessing the predictive value of (1) the number of current risky health behaviours (i.e. the current risk score), (2) the number and temporal accumulation of risky health behaviours (i.e. the temporal risk score), (3) the current and temporal accumulation together, and 4) the temporal accumulation of each health behaviour separately (i.e. the temporal risk score for each behaviour). Finally, all models were repeated with the interaction effect of age and the risk score in question to assess age-related variation in the associations. Results from the linear multilevel models are presented as unstandardized beta coefficients (B) and 95% confidence intervals (CI).

All statistical analyses were conducted with SPSS 28.0.1.1. (Armonk, NY: IBM Corp). Data were visualized with RStudio 2023.2.1. (Boston, MA).

## Results

At ages 27, 36, 42, 50, and 61, women comprised 48%, 48%, 47%, 47%, and 52% of the participants, respectively. Among the participants, 22% attained a vocational course or less as their highest education in adulthood, 38% attained vocational school education, 26% attained a bachelor’s level education, and 15% attained a university-level education.

The descriptive statistics for risky health behaviours and outcomes are shown in Supplementary Table S5. The proportion of current smokers decreased over time from almost half at age 27 to less than a fifth at age 61. Heavy alcohol consumption increased from ages 27–42 to ages 50 and 61. The proportion of physical inactivity decreased over time. There were no statistically significant differences in depressive symptoms and psychological well-being between age phases. Self-rated health was statistically significantly higher in the earlier age phases, while the number of metabolic risk factors increased over time.

**Figure 2.**
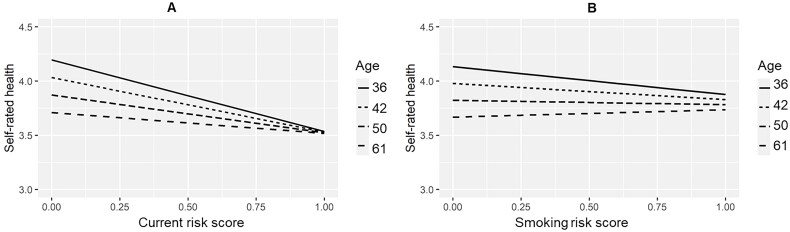
The interactions between the current risk score and age (A) and smoking risk score and age (B) on self-rated health. The current risk score indicates the number of current risky behaviours (0 = no current risky behaviours to 1 = all three current risky behaviours, i.e. smoking, heavy alcohol consumption and physical inactivity). The smoking risk score indicates the temporal accumulation of smoking risk over time by a certain age (0 = no smoking in the current and previous age phases to 1 = smoking in current and all previous age phases).

The multilevel models are presented in Tables 1 and 2 and models with interaction terms in supplementary materials (Tables S6 and S7). There were no interactions with age in any models, indicating consistent associations across ages 36, 42, 50, and 61, except for the associations between the number of current risky behaviours and self-rated health (the interaction term between age * a risk score *B* = 0.16, 95% CI 0.003–0.31) as well as between the temporal score of smoking and self-rated health (age* smoking *B* = 0.11, 95% CI 0.01–0.21). The interactions indicate that the associations of both the number of current risky behaviours and the cumulative association of smoking with self-rated health are stronger at a younger age than later in midlife (Figure 2).

**Table 2.** The associations of temporal accumulation of risky health behaviors separately with mental well-being and health.

	Depressive symptoms		Psychological well-being		Self-rated health		Metabolic risk score	
	B	95%CI	B	95%CI	B	95%CI	B	95%CI
Smoking	**0.15**	0.06–0.24	**−0.08**	−0.15– −0.01	−0.15	−0.33–0.03	0.10	−0.28–0.47
Heavy alcohol consumption	**0.21**	0.05–0.37	−0.06	−0.18–0.07	**−0.62**	−0.95– −0.28	**1.03**	0.35–1.72
Physical inactivity	0.03	−0.08–0.14	−0.00	−0.09–0.09	**−0.31**	−0.54– −0.08	**0.89**	0.37–1.42
Gender	**0.14**	0.06–0.23	0.01	−0.06–0.08	**−0.19**	−0.36– −0.03	**−0.47**	−0.79– −0.16
Education	**−0.06**	−0.1– −0.01	**0.08**	0.04–0.11	**0.08**	0–0.17	−0.05	−0.22–0.12
Age	0.01	−0.01–0.03	−0.01	−0.02–0.01	**−0.12**	−0.16– −0.08	**0.73**	0.65–0.82

Unstandardized estimates (B) and their 95% confidence intervals (CI) are presented. Bolded estimates p < 0.05. Gender: Reference group men. Number of cases for the analysis: N = 901 for depressive symptoms, N = 927 for psychological well-being, N = 1037 for self-rated health, N = 532 for metabolic risk factors.

Model 1 in Table 1 shows that having all three current risky behaviours, compared to having none, is associated with an increase of 0.10 in depressive symptoms and 0.53 in the metabolic risk score, and with a decrease of 0.10 in psychological well-being and 0.45 in self-rated health. Temporal accumulation of risky behaviours (Model 2) has a stronger association: having all three risky behaviours in the current and all previous data collection phases was associated with an increase of 0.38 in depressive symptoms and 1.49 in the metabolic risk score, and a decrease of 0.14 in psychological well-being and 0.45 in self-rated health.

Model 3 in Table 1 shows that when the number of current risky behaviours and the number of risky behaviours accumulated over time were analyzed in the same model, only the temporal risk score was statistically significantly associated with an increase in depressive symptoms and metabolic risk factors, as well as a decrease in self-rated health. Neither the number of current risky behaviours nor their accumulation over time remained statistically significantly associated with psychological well-being.

Table 2 shows that when smoking, heavy alcohol consumption, and physical inactivity were analyzed separately in the same model, smoking in the current and all previous data collection phases, compared to non-smoking in all phases, was associated with an increase of 0.15 in depressive symptoms and a decrease of 0.08 in psychological well-being. Heavy alcohol consumption in the current and all previous data collection phases was associated with an increase of 0.21 in depressive symptoms and 1.03 in the metabolic risk score, as well as a decrease of 0.62 in self-rated health. Physical inactivity in the current and all previous data collection phases, compared to being physically active in all phases, was associated with a decrease of 0.31 in self-rated health and an increase of 0.89 in the metabolic risk score.

## Discussion

The present study aimed to investigate how the number of current risky health behaviours and their temporal accumulation are associated with mental well-being and health over decades from early adulthood to the beginning of late adulthood. Having both more current risky behaviours and their accumulation over time were associated with poorer mental well-being, lower self-rated health, and a higher number of metabolic risk factors. The accumulated risk over time had greater predictive value than the number of current risky behaviours. Regarding the temporal accumulation of different risky health behaviours, smoking, heavy alcohol consumption, and physical inactivity had partly different associations: while exposure to smoking was associated with lower mental well-being, heavy alcohol consumption, and physical inactivity were associated with poorer metabolic health and self-rated health, and heavy alcohol consumption was also associated with a higher number of depressive symptoms.

The results are in line with previous studies suggesting that both the number of risky behaviours [10,11,13,14] and their temporal accumulation [10,14,15] may increase the risk for functional limitations. The results are also in line with previous findings suggesting that temporal accumulation of risky behaviours over time increases the risk for unfavourable outcomes compared to having risk only at one time point [10,14,15]. While previous longitudinal studies have mainly focused on risky health behaviours during midlife (>40 years of age) with around 20 years of follow-up time, this study extended the cumulation of risky health behaviours to early adulthood (age 27) with over 30 years of follow-up. According to former results of this longitudinal study, the formation of health behaviours begins already in childhood [43], and the age of initiation of drinking is related to drinking habits in midlife [46]. Health behaviours also have relatively high stability during midlife [6]. These findings highlight the importance of tackling risky health behaviours as early as possible to prevent their temporal accumulation which can otherwise lead to poor mental well-being and health later in life.

The findings of the present study suggest that the associations were mainly similar across time from age 36 to 61. Thus, the cumulative association of earlier risky behaviours exists already at age 36 and not only in the later phases of midlife. While risky health behaviours in midlife have been previously associated with unfavourable health outcomes in old age [10,14,15], these associations exist already earlier in adulthood. There were interactions indicating that the number of current risky behaviours and the temporal risk score of smoking play more role in self-rated health in early midlife compared to later age phases. This finding may be related to age differences in the evaluation of self-rated health: younger adults, who are more likely to be free of diseases, may base their evaluation more on health behaviours, particularly smoking, than older ones [51].

This study suggests that risky health behaviours are relevant not only for subjective and objective health outcomes but also for mental well-being. The results align with previous studies indicating that combinations of health behaviours are linked to both depressive symptoms [22] and positive aspects of mental well-being [20]. Interestingly, the temporal accumulation of risky health behaviours was particularly associated with depressive symptoms in the present study. The life course perspective should be taken into account in the development of depressive symptoms and depression in adulthood [52], and these results suggest that the accumulation of risky health behaviours over time may also be one of the important factors when preventing depressive symptoms and depression. However, the associations were relatively small as having all three risky behaviours over time was associated with an increase of 0.38 points in depressive symptoms. This is in line with studies suggesting that lifestyle interventions targeting multiple health behaviours have a small effect on depressive symptoms [53].

The analysis investigating the three risky health behaviours separately suggested that there may be partly different risky behaviours leading to these temporally cumulative associations. For example, smoking and heavy alcohol consumption were associated with lower mental well-being. The temporal accumulation of smoking was associated with both negative and positive aspects of mental well-being, i.e. with higher depressive symptoms and lower psychological well-being. This finding is in line with previous studies suggesting clear positive associations between smoking and depression [19,21,54] and also negative associations between smoking and positive aspects of mental well-being [55]. While the causal direction of this association remains unclear, it is suggested that while smoking may be used to reduce negative feelings, it is likely to increase them with withdrawal symptoms [56]. Similarly to smoking, heavy alcohol consumption may be related to stressful life circumstances and in turn cause more problems (e.g. issues with family and friends, and finances), which then expose them to depressive symptoms [57].

Both objective (metabolic risk factors) and subjective (self-rated health) were predicted by temporal accumulation of alcohol consumption and physical inactivity. These findings support the previous evidence on the associations between heavy alcohol consumption [58,59] and physical inactivity [60] with metabolic syndrome and health in general. Previous findings suggest that while the association of alcohol consumption with self-rated health may be explained by health issues, maintaining or improving physical activity over time may prevent from typical age-related decline in self-rated health [61]. An important addition to the evidence from the present study was that these associations exist already in early midlife.

In the present follow-up study, the focus was on predicting mental well-being and health with cumulating health behaviours, and the other direction was not investigated. There are likely complex bidirectional associations between health behaviours and outcomes of the present study. For example, according to the Health Belief Model, both the perceived susceptibility and severity of the negative health outcome affect the likelihood of changing health behaviours to prevent or treat the negative outcome [62,63]. Diagnosis of metabolic risk factors will likely awaken perceived susceptibility, which in turn may contribute to health behaviour change. In addition, mental well-being may both result from and influence health behaviours. For example, depressive symptoms may predict risky health behaviours [19,64] while good mental well-being may be a resource for smoking cessation [55]. These bidirectional interactions suggest that the relationships between health behaviours and health and mental well-being outcomes are not linear, but rather dynamic and potentially reciprocal.

In the current study, this complexity may have influenced the results, as health behaviours and health and mental well-being are likely to interact in a feedback loop, where each influences the other over time. For example, participants with better health and mental well-being may have been more inclined to maintain healthy behaviours, which in turn could further enhance their health and mental well-being. Conversely, those experiencing poor health and mental well-being may have been more likely to engage in risky behaviours, leading to worsened health outcomes and further declines in mental well-being. It is not possible to draw firm conclusions on causal relationships from the current observational study. Additionally, genetic, environmental, and social factors that may contribute to the associations of health behaviours with health and mental well-being were not considered in the present study. There are also many external factors and life events that may have contributed to both health behaviours and outcomes during the study period. These factors were not accounted for in the present study but provide an interesting area for future research. Further research is needed to understand the complex temporal associations between health behaviours, mental well-being, and health throughout the life course, as well as the role of aforementioned external factors and life events in these associations. Longitudinal and experimental designs help to disentangle these complex associations and provide a more comprehensive understanding of how health behaviours and mental well-being interact over time.

In comparison to previous studies that also examined other health behaviours, such as sleeping and nutrition [10,11,13,14], this study was limited to three health behaviours: smoking, alcohol consumption, and physical inactivity. While it would be important to consider health behaviours more broadly in future studies, the results of the present study suggest that focusing on these three behaviours would provide significant health benefits. Health behaviours may share similar motivational determinants, which provides an opportunity for targeting them within the same intervention [65,66]. However, it may be more effective to target smoking sequentially rather than simultaneously with other health behaviours [8]. Recent studies suggest that digital health interventions may offer a new, cost-effective way to address multiple health behaviours and reduce the risk of metabolic syndrome and other chronic conditions [67]. The results of the present study indicate that interventions should target young adults to promote healthy behaviours and prevent the accumulation of risky behaviours over adulthood. Tailored and multicomponent interventions using an ecological approach, which involves multiple levels and various stakeholders, are recommended for young adults [68]. In midlife, people may have different barriers and facilitators for health behaviours than younger or older adults [69]. For example, middle-aged smokers are likely to have maintained their habit despite various public health campaigns and need age-tailored actions emphasizing the benefits of quitting in midlife [70].

There were some limitations related to health behaviour assessments in this study. First, as mentioned above, this study was limited to three health behaviours: smoking, alcohol consumption, and physical inactivity. It is unfortunate that no information on other important health behaviours, such as nutrition, sleep, and dental hygiene, was available. As every risky behaviour is likely to increase the risk of detrimental health outcomes [9,18], the inclusion of more health behaviours could have provided even stronger associations between risk scores and health outcomes. Including more health behaviours in future studies would provide a more comprehensive outlook on participants’ behavioural risk factors. Second, only a crude estimate of each risky behaviour was used, and categorization of behaviours as risky vs. no risky excludes important information compared to continuous variables. For example, a categorization of current vs. non-current smokers was used in the study instead of more detailed information on actual cigarette exposure (e.g. pack-years), and alcohol consumption just below the risk threshold may still be detrimental to health compared to lower consumption. Categorization reduces precision and may be less sensitive to changes over time (e.g. a decrease in smoking while still remaining a current smoker). The associations between risky health behaviours and health outcomes may be stronger with more nuanced variables. However, there is short-term variation that does not last long, while health behaviours change slowly. Small nuances might not produce valid and valuable information in the long run. Third, it is also noteworthy that in the risk scores calculated for the present study, each risky behaviour had the same weight (e.g. being physically active less often than once a week vs. current smoking). The use of these crude estimates of risky behaviours with the same weight for each behaviour may oversimplify the complex associations. Applying weighting for the risk associated with each behaviour could produce different results. Since the risks associated with behaviours vary across outcomes, distinct weightings would be required for each variable. This methodology should be considered in future studies. Fourth, there were minor changes and improvements in health behaviour assessments between age phases. Future studies are recommended to assess health behaviours in more detail (e.g. using accelerometers for physical activity assessments and asking about pack-years of smoking) and include other health behaviours. Fifth, all health behaviours and outcomes were assessed through self-reports. Despite the risk of biases (e.g. recall and social desirability biases), they offer a practical and time-efficient way to gather data, especially in longitudinal studies. While this study used the best available options at the time of the data collection and repeated the same assessments over time, future studies will have more options, such as accelerometers for physical activity assessments. However, self-reports remain the only way to assess some health behaviours, such as smoking and alcohol consumption, if the interest is in risky behaviours before the existence of problem usage or other health problems registered by health care professionals. Self-reports are also the most appropriate method for assessing subjective experiences, such as feelings of well-being or depressive symptoms.

The generalizability of these results is subject to certain limitations. The sample represents the respective Finnish age cohort born in 1959 relatively well and the results are likely to be generalizable to this age group in Finland and other Western countries [30,31]. However, this cohort differs from later-born cohorts in many ways, and the overall cultural and social environment has changed over the decades. For example, health education became an independent school subject in Finland in 2004 [71] and current young adults have received more structured health education than previous cohorts. While smoking is the only risky health behaviour showing a globally decreasing trend due to changes in attitudes and restrictions [1], the use of other drugs (e.g. cannabis) has increased [72]. Thus, future generations may exhibit somewhat different risky health behaviours compared to the cohort in the current study. However, the strength of the long follow-up is to facilitate understanding of the risk behaviour of people over decades.

The Finnish participants may also have certain characteristics that differ from those of other Western countries, limiting the generalizability of the findings to the other populations. For example, both smoking and alcohol consumption have decreased over the last decades in Finland and are now lower than the EU average [73]. Despite cultural and temporal differences in the level of risky health behaviours, their associations with health outcomes are likely to remain relatively consistent across different populations (e.g. 74]. While many correlates of mental well-being are considered universal, some may vary across cultures or ethnic groups [75,76]. Consequently, the associations between risky health behaviours and mental well-being observed in this Finnish cohort may differ in other populations or cultural contexts. To strengthen the generalizability of our findings, it would be important to replicate this study with diverse samples, including different age groups and populations from various cultural backgrounds.

It should be noted that the sample was relatively small, and some nonsignificant findings may be due to the lack of statistical power. Since the prevalence of certain risky health behaviours, particularly heavy alcohol consumption earlier in midlife and physical inactivity later in midlife, was relatively low, a larger sample would provide more statistical power to estimate the associations between risky behaviours and health outcomes. For example, the interactions between age and risk scores may require a larger sample to achieve statistical significance. Future longitudinal studies should target large enough initial samples to maintain an adequate sample size despite the natural attrition in longitudinal research and enhance the findings’ generalizability. One reason for attrition is that some early deaths may be related to health problems and risky behaviours, and this needs to be taken into account when interpreting the results of the present study. Longitudinal multilevel models were used to utilize the maximum amount of available data which also increased the number of analyzed cases in the data compared to estimating separate models for each age phase. Moreover, the sample is from a unique longitudinal study including repeated measures of health behaviours and outcomes over decades from early adulthood to the beginning of late adulthood [30,31]. There was no initial attrition and attrition has remained relatively low throughout the longitudinal study [30,31]. The longitudinal study provided the opportunity to investigate the temporal accumulation of risky health behaviours over 30 years from early adulthood (age 27) to the beginning of late adulthood (age 61). The strengths of this study included not only its longitudinal design but also the multidimensional assessment of both subjective and objective health as well as positive and negative aspects of mental well-being.

In conclusion, the results indicate that both the number of current risky health behaviours and particularly their temporal accumulation are associated with poorer mental well-being, lower self-rated health, and a higher number of metabolic risk factors. While different health behaviours are important to consider together, smoking, alcohol consumption, and physical inactivity are likely to be related to partly different aspects of mental well-being and health: alcohol consumption is associated with most outcomes, smoking with poorer mental well-being, and physical inactivity with poorer health. The findings suggest that risky health behaviours are important to be considered already in early adulthood and midlife to prevent health risks occurring later in old age.

## Supplementary Material

SupplementaryMaterials.docx

## Data Availability

The Finnish law dictates that the data cannot be openly shared due to the data sensitivity and privacy of participant data. The data are stored in the Finnish Social Science Data Archive (FSD) (https://www.fsd.uta.fi/en/), except for the most recent data, which will be deposited to the FSD by the end of 2025. It is possible for those researchers who are not members or collaborators of the JYLS research team to access the pseudonymized JYLS data under certain conditions. The data access procedure follows the protocol described on the FSD website. The data analyses that support the findings of the present article are available from the corresponding author upon reasonable request.
