# Supplementary material for: Cumulative associations between health behaviours, mental well-being, and health over 30 years
Source: Ann Med. 2025 Apr 24;57(1):2479233. doi: 10.1080/07853890.2025.2479233 (PMC12024514; doi:10.1080/07853890.2025.2479233)
Supplement: SupplementaryMaterials.docx [file IANN_A_2479233_SM4658.docx]

**Cumulative associations between health behaviors, mental well-being, and health over 30 years**

**Supplementary materials**

Table S1. Smoking assessment at each age.

|  | **27** | **36 and 42** | **50 and 61** |
| --- | --- | --- | --- |
| **Question** | How often do you smoke at the moment? | Do you smoke or have you ever smoked? | Same as 36 and 42 |
| **Response options** | 1) I have never smoked: at most I have sometimes tried it, 2) I have sometimes smoked for a short period of time, 3) I do not smoke at the moment; I gave up when I was X years old, 4) less than once a week, 5) weekly but not daily, 6) daily | Same as 27 | 1) I have never smoked, 2) I have quitted smoking at age X, 3) I am currently smoking |
| **Categorization to current smokers** | 0 = No, responses 1–3  1= Yes, responses 4–6 | Same as 27 | 0 = No, responses 1–2  1= Yes, response 3 |

Table S2. Physical activity assessment at each age.

|  | **27** | **36** | **42, 50 and 61** |
| --- | --- | --- | --- |
| **Question** | How often do you do physical exercise or sport? | To what extent do you spend your leisure time for physical exercise or sport? | How often do you exercise (including incidental exercise) or pursue sports in your leisure time? |
| **Response options** | 1) not at all,  2)less than once a week,  3) about once a week,  4) 2–4 times a week,  5) almost daily | 1) not at all,  2) somewhat,  3) mainly | 1= never,  2) less than once a month,  3) 1–2 times a month,  4) once a week,  5) 2–3 times a week,  6) 4–5 times a week,  7) practically every day |
| **Categorization to physically**  **inactive** | 0= No, responses 3–5  1= Yes, responses 1–2 | 0= No, responses 2–3  1= Yes, response 1 | 0= No, responses 4–7  1= Yes, responses 1–3 |

Table S3. Alcohol consumption self-assessment with a quantity-frequency table at each age

|  | **27** | **36** | **42, 50 and 61** |
| --- | --- | --- | --- |
| **Instruction** | Here is a list of amounts of alcohol use presented. Please, estimate, how often you are used to use each amount of alcohol. Please, circle the right alternative in each question. | How much alcohol do you take at one time? If you have quit, please refer to the situation before you had quit. Circle the most appropriate frequency option on each line. One portion = a bottle of beer, cider, or long drink (33 cl, 4.5%), a glass of table wine (12 cl), a glass of strong wine (8 cl), or a shot of spirits (4cl) | Same as 36 |
| **Horizontal response options for quantity of drinking (and recoding for portions of 12g alcohol)** | 1) a bottle of lager or a glass of wine (=1), 2) 2–4 bottles of lager or half a bottle of wine (=3), 3) at least 5 bottles of lager, a bottle of wine, or half a bottle of spirits (=6). | 1) one portion or less, 2) 2–4 portions, 3) 5–7 portions, 4) 8–12 portions, 5) at least 13 portions. | 1) one portion or less, 2) 2–4 portions, 3) 5–7 portions, 4) 8–12 portions, 5) at least 13 portions, 6) 14–19 portions, 7) at least 20 portions |
| **Vertical response options for frequency of drinking (recoding for yearly drinking days)** | 1) not at all (=0), 2) at most once a year (=1), 3) several times a year (=3), 4) about once a month (=12), 5) about once a week (=52), 6) several times a week (=260) | 1) not at all (=0), 2) at most twice a year (=2), 3) once every 2 months (=6), 4) once or twice a month (=18), 5) once a week (=52), 6) 2–5 times a week (=182), 7) 6–7 times a week (=338) | Same as 36 |
| **Calculation of annual alcohol consumption** | A sum of multiplying the reported quantity (portions, recoded) by the frequency (number of drinking days per year, recoded) and multiplied by 12 g | Same as 27 | Same as 27 and 36 |
| **Categorization to heavy alcohol consumption** | 0 = No, <7000 g of pure alcohol/year for women, <10 000 g for men  1= Yes, ≥ 7000 g of pure alcohol / year for women, and ≥ 10 000 g for men | Same as 27 | Same as 27 and 36 |

References: Pitkänen, T., Kokko, K., Lyyra, A.-L., & Pulkkinen, L. (2008). A developmental approach to alcohol drinking behaviour in adulthood: A follow-up study from age 8 to age 42. *Addiction*, *103*(s1), 48–68. https://doi.org/10.1111/j.1360-0443.2008.02176.x; Pitkänen, T., Lyyra, A.-L., & Pulkkinen, L. (2005). Age of onset of drinking and the use of alcohol in adulthood: A follow-up study from age 8–42 for females and males. *Addiction*, *100*(5), 652–661. https://doi.org/10.1111/j.1360-0443.2005.01053.x

Table S4. Metabolic risk factors assessment at each age.

|  | **42** | **51** | **61** |
| --- | --- | --- | --- |
| **Metabolic risk factors** | The participants were classified as having a particular risk factor based on the following thresholds: Blood pressure ≥130/85 mmHg, triglycerides $\geq$1.7 mmol/l, plasma glucose $\geq$6.1 mmol/l, waist circumstance $>$88 cm for women and $>$102 cm for men, and HDL cholesterol $<$1.29 mmol/l for women and $<$1.04 mmol/l for men. If the participants had blood pressure, dyslipidemia, and/or diabetes medication, they were classified as having that risk factor at the current and later time points, even if the values were within thresholds/normalized afterward. | | |
| **Blood pressure** | Measured twice using a standard mercury sphygmomanometer in a sitting position after 15-min rest, measurement taken from the right arm, to the nearest 2 mm HG. The 2^nd^ measurement was used in the analyses. | Automatic sphygmomanometer was used, otherwise same as 42 | Same as 50 |
| **Waist circumference** | Measured midway between the lowest rib margin and the iliac crest. | Same as 42 | Same as 42 |
| **Blood**  **samples** | Blood sample was determined by enzymatic methods using an automatic analyzer equipment (Hitachi 917, Tokyo, Japan) | Blood sample was determined by enzymatic methods using an automatic analyzer equipment (Modular P800, Tokyo, Japan) and reagents from Roche, Mannheim, Germany | Blood sample was determined by enzymatic methods using an automatic analyzer equipment (Konelab, Vantaa, Finland) and reagents from Thermo Fisher Scientific, Vantaa, Finland |
| **Triglycerides** | Reagents from Roche, Basel, Switzerland (TG GPO-PAP Kit No. 1730711) | TG, Triglycerides GPO-PAP, Cobas Cat.No. 11730711 216 | Triglycerides Kit No. 981786 |
| **HDL**  **cholesterol** | Reagents from Boehringer, Mannheim, Germany (HDL/LDL-C Plus Kit No. 1930648) | HDL-C plus 3rd generation, HDL-Cholesterol, no pretreatment, Cobas Cat.No. 04713214 190 | HDL-Cholesterol Kit No. 981955 |
| **Blood glucose** | Reagents from Boehringer, Mannheim, Germany (GLU Kit No. 1876899) | GLU, Gluco-quant®Glucose/HK, Cobas Cat.No. 11876899 216 | Glucose (HK) Kit No. 981779 |

References: Kinnunen, M.-L., Kokkonen, M., Kaprio, J., & Pulkkinen, L. (2005). The associations of emotion regulation and dysregulation with the metabolic syndrome factor. *Journal of Psychosomatic Research*, *58*(6), 513–521. https://doi.org/10.1016/j.jpsychores.2005.02.004; Kinnunen, M.-L., Metsäpelto, R.-L., Feldt, T., Kokko, K., Tolvanen, A., Kinnunen, U., Leppänen, E., & Pulkkinen, L. (2012). Personality profiles and health: Longitudinal evidence among Finnish adults. *Scandinavian Journal of Psychology*, *53*(6), 512–522. https://doi.org/10.1111/j.1467-9450.2012.00969.

Table S5. Proportions of risky health behaviors and descriptives for mental well-being and health outcomes at each age

|  | 27 | | 36 | | 42 | | 50 | | 61 | | Age  difference* |
| --- | --- | --- | --- | --- | --- | --- | --- | --- | --- | --- | --- |
|  | n | % | n | % | n | % | n | % | n | % |  |
| Current smoker | |  |  |  |  |  |  |  |  |  | All** |
| Yes | 160 | 49.1 | 111 | 35.7 | 121 | 42.5 | 77 | 28.7 | 37 | 18.0 |  |
| No | 166 | 50.9 | 200 | 64.3 | 163 | 57.5 | 191 | 71.3 | 169 | 82.0 |  |
| Heavy alcohol consumption |  |  |  |  |  |  |  |  |  |  | 27<36–61  36,42< 50,61 |
| Yes | 10 | 3.1 | 33 | 11.7 | 33 | 11.8 | 49 | 18.4 | 33 | 16.0 |  |
| No | 326 | 96.9 | 273 | 88.3 | 247 | 88.2 | 218 | 81.6 | 173 | 84.0 |  |
| Physical inactivity | |  |  |  |  |  |  |  |  |  | 27,36>42–61 42>50 |
| Yes | 100 | 31.2 | 95 | 30.7 | 47 | 16.9 | 37 | 14.2 | 21 | 10.2 |  |
| No | 221 | 68.8 | 214 | 69.3 | 231 | 83.1 | 224 | 85.8 | 185 | 89.8 |  |
|  |  |  | M | SD | M | SD | M | SD | M | SD |  |
| Depressive symptoms | | | 1.45 | 0.35 | 1.51 | 0.45 | 1.45 | 0.35 | 1.46 | 0.37 | ns. |
| Psychological well-being | | | 3.18 | 0.32 | 3.14 | 0.34 | 3.18 | 0.33 | 3.18 | 0.33 | ns. |
| Self-rated health | |  | 4.13 | 0.91 | 3.80 | 0.80 | 3.72 | 0.82 | 3.85 | 0.83 | 36>42,50, 61; 42>50 |
| Metabolic risk factors | | |  |  | 1.37 | 1.23 | 1.86 | 1.34 | 2.74 | 1.45 | 42<50,61; 50<61 |

*Age differences were tested with the McNemar test for binary variables and with paired samples t-test for continuous variables. If p<0.05, the difference is reported in the table. **Statistically significant difference between all age groups. For depressive symptoms and metabolic risk factors, a higher number indicates more symptoms/risk factors. For psychological well-being and self-rated health, a higher number indicates better psychological well-being and self-rated health.

Table S6. The models including interaction term between time and risk score.

|  | Depressive  symptoms | | Psychological  well-being | | Self-rated health | | Metabolic  risk score | |
| --- | --- | --- | --- | --- | --- | --- | --- | --- |
|  | B | 95%CI | B | 95%CI | B | 95%CI | B | 95%CI |
| **Model 1** |  |  |  |  |  |  |  |  |
| Current risk score | 0.11 | -0.06–0.28 | -0.07 | -0.21–0.07 | **-0.82** | -1.23– -0.40 | 0.25 | -0.86–1.36 |
| Gender | **0.11** | 0.03–0.29 | 0.01 | -0.05–0.08 | -0.14 | -0.3–0.02 | **-0.54** | -0.85– -0.22 |
| Education | **-0.07** | -0.11– -0.03 | **0.08** | 0.05–0.12 | **0.10** | 0.02–0.18 | -0.11 | -0.28–0.06 |
| Age | 0.01 | -0.01–0.03 | 0.00 | -0.02–0.01 | **-0.16** | -0.21– -0.12 | **0.70** | 0.60–0.80 |
| Age *  risk score | -0.01 | -0.07–0.06 | -0.01 | -0.07–0.04 | **0.16** | 0.003–0.31 | 0.10 | -0.27–0.47 |
| **Model 2** |  |  |  |  |  |  |  |  |
| Temporal risk score | **0.35** | 0.11–0.59 | -0.06 | -0.25–0.13 | **-1.02** | -1.57– -0.47 | 0.77 | -0.76–2.30 |
| Gender | **0.13** | 0.04–0.21 | 0.02 | -0.05–0.09 | -0.16 | -0.32–0.002 | **-0.47** | -0.79– -0.15 |
| Education | **-0.05** | -0.1– -0.01 | **0.07** | 0.04–0.11 | **0.09** | 0.001–0.17 | 0.01 | -0.16–0.18 |
| Age | 0.01 | -0.01–0.04 | 0.001 | -0.02–0.02 | **-0.15** | -0.2– -0.09 | **0.68** | 0.55–0.82 |
| Age *  risk score | 0.02 | -0.07–0.10 | -0.04 | -0.11–0.02 | 0.10 | -0.1–0.30 | 0.28 | -0.24–0.80 |

Unstandardized estimates (B) and their 95% confidence intervals (CI) presented. Bolded estimates p<0.05. Gender: Reference group men.

Table S7. The models including interaction term between time and risk score.

|  | Depressive  symptoms | | | | Psychological  well-being | | | | Self-rated health | | | | Metabolic  risk score | |
| --- | --- | --- | --- | --- | --- | --- | --- | --- | --- | --- | --- | --- | --- | --- |
|  | B | | 95%CI | | B | | 95%CI | | B | | 95%CI | | B | 95%CI |
| **Smoking** | |  | |  | |  | |  | |  | |  |  |  |
| Smoking | **0.14** | | 0.02–0.25 | | -0.04 | | -0.13–0.05 | | **-0.37** | | -0.63– -0.10 | | -0.34 | -1.02–0.35 |
| Alcohol | **0.21** | | 0.05–0.37 | | -0.04 | | -0.17–0.09 | | **-0.66** | | -1.00– -0.33 | | **0.97** | 0.28–1.66 |
| Inactivity | 0.03 | | -0.08–0.14 | | 0.00 | | -0.09–0.09 | | **-0.31** | | -0.54– -0.08 | | **0.90** | 0.38–1.42 |
| Gender | **0.14** | | 0.06–0.23 | | 0.01 | | -0.06–0.08 | | **-0.19** | | -0.36– -0.03 | | **-0.48** | -0.79– -0.16 |
| Education | **-0.06** | | -0.10– -0.01 | | **0.08** | | 0.04–0.11 | | **0.08** | | 0.00–0.17 | | -0.05 | -0.22–0.12 |
| Age | 0.01 | | -0.01–0.03 | | 0.00 | | -0.02–0.02 | | **-0.16** | | -0.21– -0.11 | | **0.69** | 0.58–0.79 |
| Age*Smoking | 0.01 | | -0.03–0.05 | | -0.02 | | -0.06–0.01 | | **0.11** | | 0.01–0.21 | | 0.17 | -0.06–0.40 |
| **Alcohol** |  | |  | |  | |  | |  | |  | |  |  |
| Smoking | **0.15** | | 0.06–0.24 | | **-0.08** | | -0.15– -0.01 | | -0.15 | | -0.33–0.03 | | 0.10 | -0.28–0.47 |
| Alcohol | **0.28** | | 0.03–0.54 | | 0.04 | | -0.17–0.25 | | **-0.78** | | -1.37– -0.19 | | 0.54 | -0.95–2.04 |
| Inactivity | 0.03 | | -0.08–0.14 | | 0.00 | | -0.09–0.09 | | **-0.31** | | -0.54– -0.08 | | **0.90** | 0.38–1.43 |
| Gender | **0.14** | | 0.06–0.23 | | 0.01 | | -0.06–0.08 | | **-0.19** | | -0.36– -0.03 | | **-0.47** | -0.79– -0.16 |
| Education | **-0.06** | | -0.10– -0.01 | | **0.08** | | 0.04–0.11 | | **0.08** | | 0.00–0.17 | | -0.05 | -0.21–0.12 |
| Age | 0.01 | | 0–0.03 | | 0.00 | | -0.02–0.01 | | **-0.13** | | -0.17– -0.08 | | **0.72** | 0.63–0.82 |
| Age*Alcohol | -0.03 | | -0.11–0.05 | | -0.04 | | -0.11–0.03 | | 0.07 | | -0.14–0.27 | | 0.18 | -0.30–0.65 |
| **Physical inactivity** |  | |  | |  | |  | |  | |  | |  |  |
| Smoking | **0.15** | | 0.06–0.24 | | **-0.08** | | -0.15– -0.01 | | -0.15 | | -0.33–0.03 | | 0.10 | -0.28–0.47 |
| Alcohol | **0.21** | | 0.05–0.37 | | -0.06 | | -0.18–0.07 | | **-0.62** | | -0.95– -0.29 | | **1.03** | 0.34–1.72 |
| Inactivity | 0.02 | | -0.12–0.16 | | 0.00 | | -0.12–0.11 | | -0.32 | | -0.65–0.01 | | **0.55** | -0.41–1.50 |
| Gender | **0.14** | | 0.06–0.23 | | 0.01 | | -0.06–0.08 | | **-0.19** | | -0.36– -0.03 | | **-0.47** | -0.79– -0.15 |
| Education | **-0.06** | | -0.10– -0.01 | | **0.08** | | 0.04–0.11 | | **0.08** | | 0.00–0.17 | | -0.05 | -0.22–0.12 |
| Age | 0.01 | | -0.01–0.03 | | -0.01 | | -0.02–0.01 | | **-0.12** | | -0.17– -0.07 | | **0.71** | 0.60–0.81 |
| Age*Inactivity | 0.01 | | -0.05–0.07 | | 0.00 | | -0.05–0.05 | | 0.01 | | -0.13–0.15 | | 0.15 | -0.20–0.50 |

Unstandardized estimates (B) and their 95% confidence intervals (CI) presented. Bolded estimates p<0.05. Gender: Reference group men.
